# Biotic Factors Influence Microbiota of Nymph Ticks from Vegetation in Sydney, Australia

**DOI:** 10.3390/pathogens9070566

**Published:** 2020-07-13

**Authors:** Shona Chandra, Jan Šlapeta

**Affiliations:** Sydney School of Veterinary Science, Faculty of Science, University of Sydney, Sydney, NSW 2006, Australia; shona.chandra@sydney.edu.au

**Keywords:** bacterial profile, *cox*1, *Haemaphysalis bancrofti*, *Ixodes holocyclus*, *Ixodes trichosuri*, *Ixodes tasmani*, V3-V4 *16S* rRNA gene

## Abstract

Ticks are haematophagous ectoparasites of medical and veterinary significance due to their excellent vector capacity. Modern sequencing techniques enabled the rapid sequencing of bacterial pathogens and symbionts. This study’s aims were two-fold; to determine the nymph diversity in Sydney, and to determine whether external biotic factors affect the microbiota. Tick DNA was isolated, and the molecular identity was determined for nymphs at the *cox*1 level. The tick DNA was subjected to high throughput DNA sequencing to determine the bacterial profile and the impact of biotic factors on the microbiota. Four nymph tick species were recovered from Sydney, NSW: *Haemaphysalis bancrofti*, *Ixodes holocyclus*, *Ixodes trichosuri* and *Ixodes tasmani*. Biotic factors, notably tick species and geography, were found to have a significance influence on the microbiota. The microbial analyses revealed that Sydney ticks display a core microbiota. The dominating endosymbionts among all tick species were *Candidatus* Midichloria sp. Ixholo1 and *Candidatus* Midichloria sp. Ixholo2. A novel *Candidatus* Midichloria sp. OTU_2090 was only found in *I. holocyclus* ticks (nymph: 96.3%, adult: 75.6%). *Candidatus* Neoehrlichia australis and *Candidatus* Neoehrlichia arcana was recovered from *I. holocyclus* and one *I. trichosuri* nymph ticks. *Borrelia* spp. was absent from all ticks. This study has shown that nymph and adult ticks carry different bacteria, and a tick bite in Sydney, Australia will result in different bacterial transfer depending on tick life stage, tick species and geography.

## 1. Introduction

Ticks (Acari: Ixodida) are important vectors for diseases and the application of high throughput DNA sequencing enables the study of the bacterial symbionts, commensals and pathogenic microorganisms that ticks may carry. The bacterial community within ticks have been well studied using next-generation sequencing (NGS) technologies, enabling the rapid sequencing of their microbiota and microbiome [1,2,3]. Globally, there have been studies implementing NGS that look at ticks, but few that involved a comparison between questing ticks across at least two life stages [4,5,6,7]. Such studies have found that bacterial composition diversity differs with host species, geographic distribution and tick life stage [5,8].

In Australia, the most important tick in the medical and small animal veterinary context is the Australian paralysis tick *Ixodes holocyclus* Neumann, 1899. Found along the eastern coastline, *I. holocyclus* possesses a potent neurotoxin that causes fatal paralysis eventuating in death for many domestic animals, and can have a flaccid paralysis effect in humans, particularly infants [9,10,11,12]. In humans, salivary secretions from *I. holocyclus* bites have been implicated with causing a red meat allergy (alpha-1, 3-galactose allergy) and hypersensitivity for allergic reactions [13,14]. Despite the medical and veterinary significance of ticks, there has been no systematic survey and/or study involving NGS methods to determine the diversity of the bacterial microbial communities within the common ticks which have been flagged in Sydney.

Previous studies using NGS methods have determined that the bacterial diversity between the tick life stages are different and are altered by previous life stage’s host-blood meal [4,5,8]. Studies which have compared the bacterial microbiota between adults and nymphs have indicated that nymph ticks have higher bacterial diversity compared to the adult tick life stage [4,5]. However, with few studies published in Australia, there has been a gap in scientific literature regarding the microbial comparison between Australian tick life stages [1,15]. Further, as *Borrelia* spp. have been identified in Sydney, Australia [2], it is crucial to survey Sydney’s questing nymph ticks to determine the diversity of pathogens vectored by the ticks in the area.

In Sydney, the most common tick species found in the environment is *I. holocyclus*, however, other tick species including the wallaby tick *Haemaphysalis bancrofti* Nuttall & Warburton, 1915, the bush tick *Haemaphysalis longicornis* Neumann, 1901, the common marsupial tick *Ixodes tasmani* Neumann, 1899, and the possum tick *Ixodes trichosuri* Roberts, 1960 have been anecdotally recorded [16,17]. Further, *H. bancrofti* and *H. longicornis* have been known to seek humans as a possible host species [1]. With more ticks than just *I. holocyclus* present in the environment, adult humans, children and their companion animals are potentially at risk of being exposed to more pathogens than commonly thought.

Most ticks of veterinary, medical and public health significance are three-host ticks, including *I. holocyclus* [18]. In Europe and North America, *Ixodes ricinus* Linnaeus, 1758, *Ixodes scapularis* Say, 1821 (syn. *Ixodes dammini*), and *Ixodes pacificus* Cooley & Kohls, 1943 are three-host ticks which are recognised vectors of the bacterial spirochaete, *Borrelia burgdorferi* sensu lato (s.l.), the causative agent of Lyme borreliosis [19,20,21,22]. The spirochaete is not transmitted trans-ovarially, so hatched larval ticks are free from *B. burgdorferi* s.l. [23,24]. Larvae of *Ixodes* spp. will then feed on an infected wildlife host, causing the larvae to become infected and it will moult into a nymph tick where the bacteria will undergo rapid replication [25,26]. The *Borrelia* spirochaete will actively migrate out of an infected nymph during feeding, and then moult into an adult tick with lower burdens of *B. burgdorferi* s.l. [24,26,27]. Both nymph and adult ticks can parasitise humans, but due to the biology of *B. burgdorferi* s.l. and the small size of nymph ticks, unfed nymphs are more efficient vectors and are primarily responsible for causing Lyme borreliosis [19,24,25,26,27].

The aims of this study were: to determine the nymph diversity of ticks found around the Sydney region, and to determine whether external biotic factors influence the tick’s microbiota. To do so, unfed nymphs were collected by flagging vegetation around the Northern Beaches, and North Shore areas in Sydney. Two DNA isolation methods and two *16S* ribosomal RNA (rRNA) hypervariable regions were initially compared to determine which combination afforded the greatest bacterial diversity. The nymph ticks were subject to conventional PCR and Sanger sequencing for speciation and modern NGS tools to determine their *16S* rRNA bacterial profile. The microbial contents of the tick nymphs were evaluated using the V3-V4 *16S* rRNA gene amplicon profile and compared to adult ticks collected from dogs to determine the differences between external factors, including the tick itself as a host for the bacteria, the life stages, tick species, tick host and geographic location.

## 2. Results

### 2.1. Pilot Study Determined gDNA Isolation and V3-V4 16S rRNA Gene Diversity Profiling Assay Optimal Combination for Tick Microbiota Studies

For the pilot study, six adult female *Ixodes holocyclus* (SC0063-1 to SC0063-6) of varying levels of engorgement were collected from a single dog host. The single host was important to reduce bias and variability from the host and the blood meal. As the ticks were bisected longitudinally, each half subject to different DNA isolation method (genomic versus faecal DNA isolation procedure) and *16S* rRNA gene diversity profiling assays (V1-V3 versus V3-V4 hypervariable region), it would allow for the DNA isolation biases and diversity profiling biases to be seen. We discovered there was no significant difference between the OTUs generated at either the DNA isolation method variable, or the hypervariable region selected (Supplementary Figure S1). However, while there were more OTUs generated in the V1-V3 hypervariable region, we noted that there were less unassigned OTUs in the V3-V4 hypervariable region than in the V1-V3 region. At the bacterial genus level, there were more bacteria identified at the V3-V4 hypervariable region than the V1-V3, where many bacteria could only be identified to the family level. From this, it was decided that moving forwards, we would continue with the genomic DNA (gDNA) isolation procedure and the V3-V4 hypervariable region as the optimal approach for our tick microbial study.

Morphologically, SC0063-1 to SC0063-6 were identified as *I. holocyclus* and gDNA analysis at the *cox*1 mtDNA marker confirmed that all were molecularly *I. holocyclus* (99.8% identity, 603/604 nt, AB075955). All six ticks were molecularly identical to each other, and the was a distinct A/G polymorphism at residue 310 across the 604 nt sequence from there reference *I. holocyclus cox*1 sequence (AB075955).

Both V1-V3 and V3-V4 *16S* rRNA hypervariable regions were dominated by OTU_1 belonging to *Candidatus* Midichloria sp. Ixholo1 (Supplementary Figure S1). At the class level, both hypervariable regions were dominated by Alphaproteobacteria followed by Gammaproteobacteria and Betaproteobacteria. At the order level, both hypervariable regions were dominated by Rickettsiales and Enterobacteriales (Supplementary Figure S1). However, the V1-V3 region detected Aeromondales and Campylobacterales, which was not detected by the V3-V4 region (Supplementary Figure S1). At the family level, Candidatus Midichloriaceae and Enterobacteriaceae dominated both hypervariable regions, followed by Comamonadaceae, Aeromonadaceae and Campylobacteraceae at the V1-V3 hypervariable region and Rhizobiaceae, Comamonadaceae and Oxalobacteraceae at the V3-V4 region. (Supplementary Figure S1). More genera were described at the V3-V4 hypervariable region than at the V1-V3 region, where many OTUs were only identifiable up to the family level (Supplementary Figure S1). While the two dominating bacteria were identical across both hypervariable regions, variability was seen in the non-dominant bacteria. 

At the OTU level for the V1-V3 hypervariable region, multivariate analysis by non-metric multidimensional scaling (nMDS) methods with Bray-Curtis similarity at 35% revealed clustering for the faecal and genomic DNA kits. The permutation based hypothesis testing approach, analysis of similarities (ANOSIM), revealed the OTUs generated was significantly different (R = 0.741, *p* = 0.002). At the OTU level for the V3-V4 hypervariable region, multivariate analysis by nMDS with Bray-Curtis similarity at 35% revealed clustering for the faecal and genomic DNA kits. ANOSIM revealed the OTUs generated was significantly different (R = 0.976, *p* = 0.002). Similarly, at the genus level for the V1-V3 hypervariable region, multivariate analysis by nMDS with Bray-Curtis similarity at 40% revealed clustering for the faecal and genomic DNA kits. ANOSIM revealed the OTUs generated was significantly different (R = 0.739, *p* = 0.002). At the genus level for the V3-V4 hypervariable region, multivariate analysis by nMDS with Bray-Curtis similarity at 35% revealed clustering for the faecal and genomic DNA kits. ANOSIM revealed the OTUs generated was significantly different (R = 0.978, *p* = 0.002). At the genus level, it was determined that there were 12 OTUs that were overlapped between the two *16S* rRNA hypervariable regions (V1-V3: 12/68, V3-V4: 12/63), indicating that the hypervariable regions displayed some bias towards certain genera of bacteria. Following, the Mann-Whitney Test for significance revealed there was no significant difference (*p* = 0.470) between OTUs generated for *16S* rRNA hypervariable regions, V1-V3 or V3-V4, indicating no significant difference between hypervariable regions used (Supplementary Figure S1).

### 2.2. Ixodes holocyclus, Ixodes trichosuri, Ixodes tasmani and Haemaphysalis bancrofti Nymph Ticks Recovered from Vegetation in Sydney

**Figure 1:**
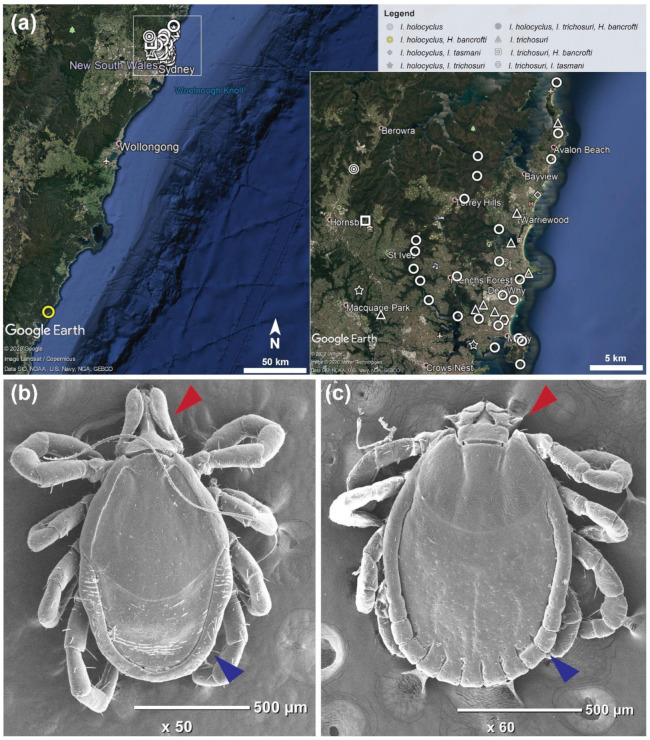
(**a**) Collection points and flagging location for nymph ticks from the eastern coastline in Australia. Zoomed-in segment depicts collection points for nymph ticks from the Northern Beaches and North Shore regions in Sydney, Australia. Map sourced from data imported into Google Earth, 2020. (**b**) Scanning electron microscopy of SC790-8 *Ixodes* spp. nymph, collected from Murramarang National Park, South Durras, NSW. Red arrow pointing to long, elongate palps; blue arrow pointing to lack of festoons, and the narrow body profile at the posterior margin. (**c**) Scanning electron microscopy of SC790-9 *Haemaphysalis bancrofti* nymph, collected from Murramarang National Park, South Durras, NSW. Red arrow pointing to short, wide palps, and palpal article 2 with large lateral projection; blue arrow pointing to prominent festoons, and the wider body profile at the posterior margin.

Nymph ticks were collected from 39 locations on the Northern Beaches and North Shore of Sydney, New South Wales (NSW) and one location from the South Coast of NSW (Figure 1a). For the nymphs, all ticks (*n* = 175) were morphologically identified to the tick genus level, which revealed *Ixodes* spp. (*n* = 164) and *Haemaphysalis* spp. (*n* = 11) was flagged from vegetation (Figure 1b,c).

**Figure 2:**
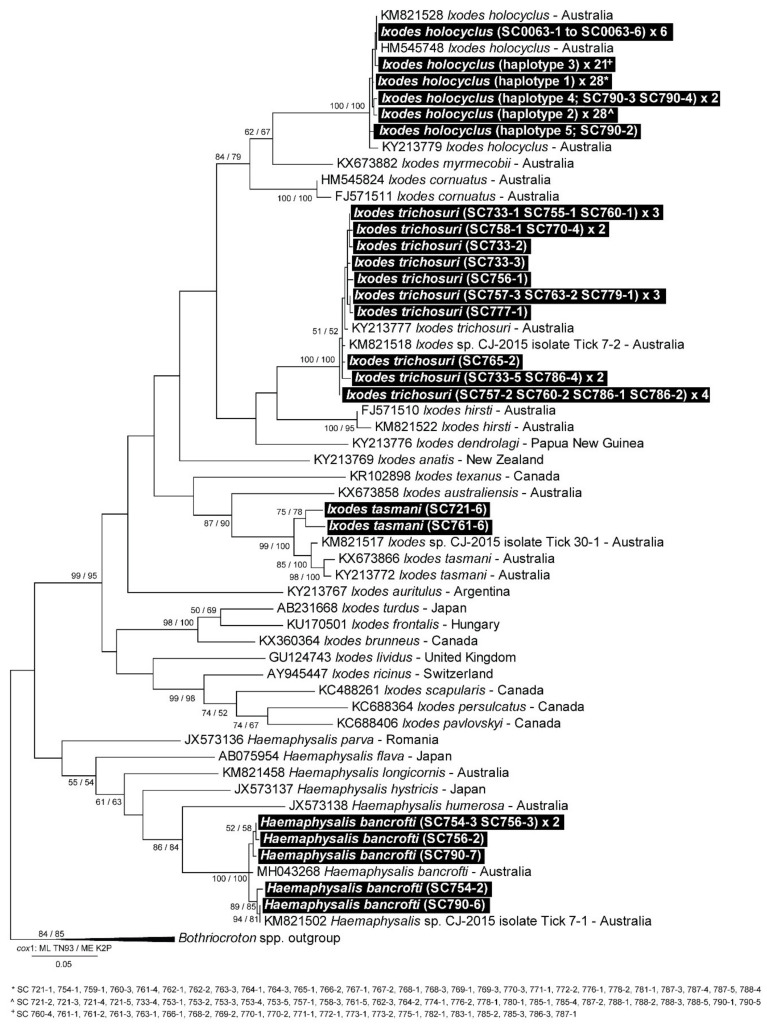
Molecular phylogenetic analysis of *Ixodes* spp. and *Haemaphysalis* spp. ticks at the cytochrome c oxidase subunit I (*cox*1) ribosomal DNA locus. The evolutionary history, evolutionary distance and evolutionary rate differences were inferred by using the following methods: maximum likelihood method based on the Tamura-Nei (ML TN93) and minimum evolution with Kimura-2 Parameters (ME K2P) models. Test of phylogeny was implicated through the Bootstrap method (1000 replications). Codon positions included were 1st and 2nd. The bootstrap confidence intervals (%) have been grouped as follows: ML TN93/ME K2P, and values < 50% have been hidden. The evolutionary analyses were conducted in MEGA 7 [28].

For more in-depth identification, 108 were selected for molecular identification to the species level at *cox*1 mtDNA region (Supplementary Table S1). Of these, it was determined that in addition to *I. holocyclus* (80/108, 99.1–100% identity, AB075955), there were *Ixodes trichosuri* Roberts, 1960 (20/108, 99–100% identity, KY213778), *Ixodes tasmani* Neumann, 1899 (2/108, 97.4–98.8% identity, MH043269) and *Haemaphysalis bancrofti* Nuttall & Warburton, 1915 (6/108, 97.9–99.8% identity, MH043268) present in the area (Figure 2).

### 2.3. Only Adult Ixodes holocyclus Ticks Recovered from Dogs in Sydney Veterinary Clinics

**Figure 3:**
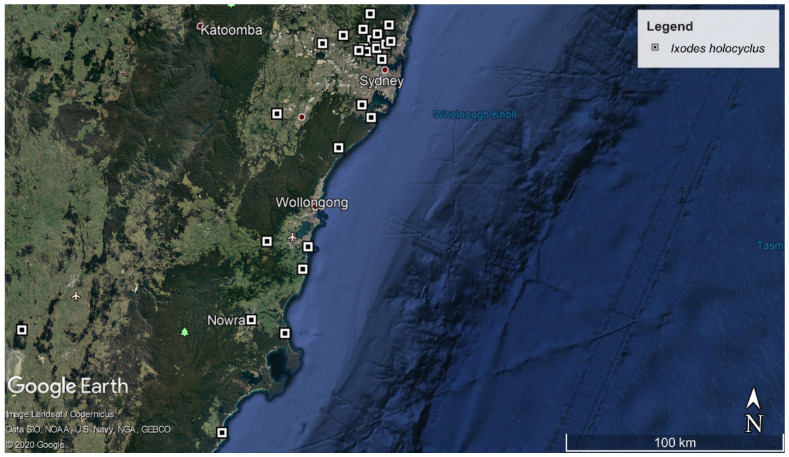
Collection points and flagging location for adult ticks from the eastern coastline in Australia. Map sourced from data imported into Google Earth, 2020.

The adult ticks recovered from veterinary clinics were from 26 distinct localities across the eastern coastline of NSW, ranging from the Northern Beaches to the South Coast (Figure 3). All specimens (*n* = 92) were weighed prior to DNA isolation (Supplementary Table S2). The ticks were morphologically identified as *I. holocyclus* and displayed the characteristic features including the leg colour pattern, the scutum proportion was wider than long, there was the notable absence of the cornua, short cervical grooves and distinct anterior position of the anal groove compared to the anus.

### 2.4. Pre-Treatment of Adult and Nymph Tick Bacterial Diversity at the 16S rRNA Hypervariable Region V3-V4

The dominating OTUs in V3-V4 bacterial profile were *Ca.* Midichloria sp. Ixholo1 (OTU_1, 69.4%), and *Ca.* Midichloria sp. Ixholo2 (OTU_1948, 21.5%). The two dominating OTUs (OTU_1, OTU_1948) were excluded, and reads belonging to phylum Proteobacteria (82.5%) dominated the V3-V4 hypervariable region. Reads in phylum Actinobacteria (12.3%), Bacteriodetes (0.6%), and Firmicutes (4.1%) were also present. Of the reads within the phylum Proteobacteria, most were of class Gammaproteobacteria (60.2%), followed by Alphaproteobacteria (33.3%) and Betaproteobacteria (6.4%). Shannon diversity indices (*H*’; t-test: t = 93.77, *p* = 0.007) and the Simpsons index (*1-λ*’; t-test: t = 48.68, *p* = 0.013) indicated that there was a significant difference between the bacterial composition in nymph and adult ticks (Table 1).

### 2.5. Presence of Candidatus Midichloria spp. in Adult and Nymph Ticks

**Figure 4:**
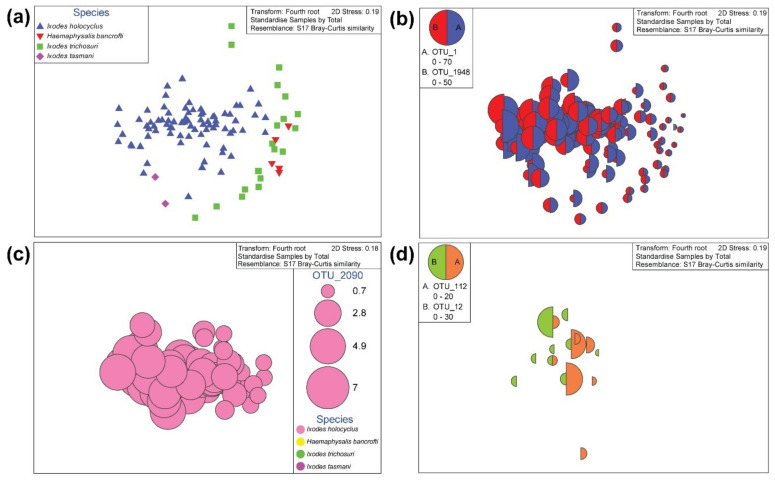
Multivariate statistical analysis of the V3-V4 bacterial profile for *Ixodes holocyclus*, *Ixodes trichosuri*, *Ixodes tasmani* and *Haemaphysalis bancrofti* nymph ticks from Sydney, Australia. Non-metric multidimensional scaling (nMDS) ordination plots for bacterial abundance and bubble plots. Outliers have been removed for the nMDS plot, and from further analyses. The outliers which were removed were: JS3368 and JS3299 (low reads). (**a**) nMDS ordination plots for bacterial abundance, shown at the operational taxonomic unit (OTU) level. (**b**) Plot for OTU_1 (*Ca*. Midichloria sp. Ixholo1) and OTU_1948 (*Ca*. Midichloria sp. Ixholo2) reveal that these endosymbionts are prevalent across all four nymph tick species collected. (**c**) Plot for OTU_2090 (97.3% identity to *Ca*. Midichloria sp. Ixholo2 (FM992373)) reveal that this endosymbiont is unique to *Ixodes holocyclus* nymph ticks. (**d**) Plot for OTU_12 (*Candidatus* Neoehrlichia arcana) and OTU_112 (*Candidatus* Neoehrlichia australis) reveal that these endosymbionts were mostly present in *Ixodes holocyclus*, except for one *Ixodes trichosuri* nymph tick (JS3333).

In nymph ticks, the nMDS ordination plots for bacterial abundance, shown at the OTU level reveal an apparent clustering among the four tick species (Figure 4a). Visualising the bubble plots for OTU_1 (*Ca.* Midichloria sp. Ixholo1) and OTU_1948 (*Ca.* Midichloria sp. Ixholo2) over the alignment of samples with nMDS distribution revealed that these two OTUs were abundant in most nymph ticks, and not just in nymphs identified as *I. holocyclus* (Figure 4b). Interestingly, a novel *Ca.* Midichloria sp. was found (OTU_2090) which had 97.3% identity to *Ca.* Midichloria sp. Ixholo2 (FM992373). A bubble plot was generated for OTU_2090, which was unique to *I. holocyclus* nymphs (77/80, 96.3%), and was absent from the other nymph species found (Figure 4c). The bubble plot generated for OTU_12 (*Candidatus* Neoehrlichia arcana) and OTU_112 (*Candidatus* Neoehrlichia australis) revealed that these OTUs were only present in *I. holocyclus*, with the exception of one *I. trichosuri* (JS3333; OTU_112) (Figure 4d).

**Figure 5:**
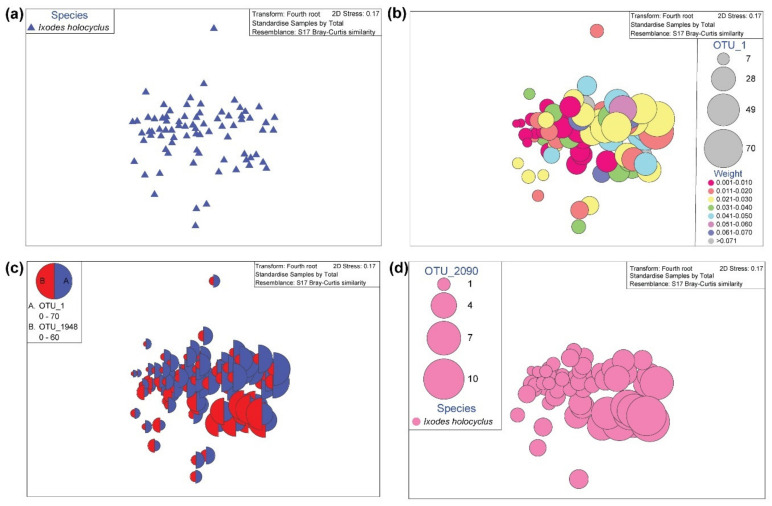
Multivariate statistical analysis of the V3-V4 bacterial profile for adult *Ixodes holocyclus* ticks from Sydney, Australia. Non-metric multidimensional scaling (nMDS) ordination plots for bacterial abundance, bootstrap averages plot and bubble plots. (**a**) nMDS ordination plots for bacterial abundance, shown at the operational taxonomic unit (OTU) level. (**b**) Bubble plot for OTU_1 (Ca. Midichloria sp. Ixholo1) with the weight factor overlayed. (**c**) Bubble plot for OTU_1 (*Ca*. Midichloria sp. Ixholo1) and OTU_1948 (*Ca*. Midichloria sp. Ixholo2) reveal that these endosymbionts are prevalent across the *Ixodes holocyclus* adult tick specimens. (**d**) Bubble plot for OTU_2090 (97.3% identity to Ca. Midichloria sp. Ixholo2 (FM992373)) reveal that this endosymbiont is present in most *Ixodes holocyclus* adult ticks.

In adult ticks, the nMDS ordination plot for bacterial abundance, shown at the OTU level (Figure 5a) display a clustering of OTUs generated in adult *I. holocyclus* ticks. Plotting bubble plots for OTU_1 (*Ca*. Midichloria sp. Ixholo1) over the weight factor indicates there is little relationship with weight of the tick and abundance of OTU_1 (Figure 5b). Plotting bubble plots for OTU_1 (*Ca.* Midichloria sp. Ixholo1 and OTU_1948 (*Ca.* Midichloria sp. Ixholo2) over the alignment of samples with nMDS distribution revealed that these two OTUs were abundant in most adult ticks, all of which were morphologically identified as *I. holocyclus* (Figure 5c). Like in the nymph ticks, a bubble plot was generated for OTU_2090 (97.3% identity *Ca.* Midichloria sp. Ixholo2; FM992373), which was found in adult *I. holocyclus* (59/78, 75.6%) ticks (Figure 5d). There was an absence of OTU_12 (*Ca.* Neoehrlichia arcana) and OTU_112 (*Ca.* Neoehrlichia australis) in adult *I. holocyclus* specimens, despite their presence in nymphs.

To determine the bacterial diversity within the nymph ticks, we compiled the OTUs and sorted the reads into four main taxonomic ranks (class, order, family, genus) and analysed the bacterial diversity at each level. At the class level, nymph ticks in Sydney are dominated by Alphaproteobacteria, while at the order level, they are mostly dominated by Rickettsiales which were visualised on bar graphs (Supplementary Figure S2a,b). When the two dominating OTUs (OTU_1, OTU_1948; *Ca.* Midichloria spp.) were hidden from the analysis, it still revealed high bacterial diversity among nymph ticks at the bacterial family (55 taxa identified) and genus (78 taxa identified) levels, visualised on bar graphs (Supplementary Figure S2c,d).

### 2.6. Permutation-Based Hypothesis Testing Reveals External Factors Can Influence the Tick’s Microbiota

**Figure 6:**
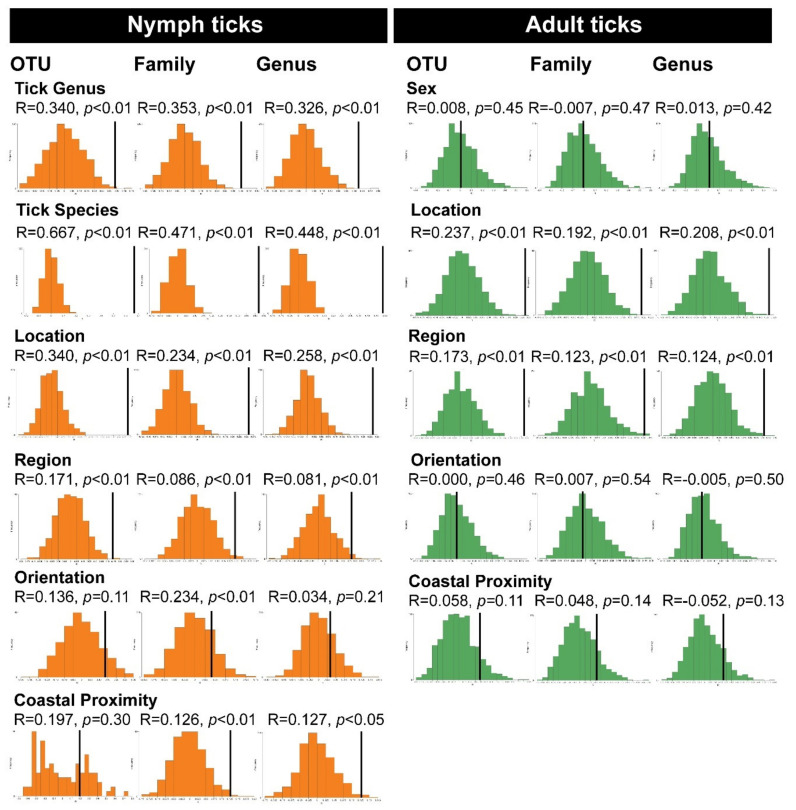
Analysis of similarities (ANOSIM) histograms for nymph *Ixodes holocyclus*, *Ixodes trichosuri*, *Ixodes tasmani* and *Haemaphysalis bancrofti* ticks (orange) and adult *I. holocyclus* ticks (green) at the bacterial OTU, family and genus levels.

Ticks species were collected from greater Sydney and eastern New South Wales (Figure 1). To determine whether the geographical location of collection has a significant effect on tick samples we evaluated it using the permutation-based hypothesis testing approach (analysis of similarities, ANOSIM) on Bray-Curtis dissimilarity matrix (Figure 6). Location was defined as the suburb of which the ticks were collected from, or where the dog host of the adult tick was living. The factor ‘region’ was defined as the NSW government’s division of state’s political and administrative regions and localities (i.e. Northern Beaches, North Shore, South Coast, etc.). The north–south orientation was determined in relation to Sydney CBD and for coastal proximity we assigned each samples with a category (1–5) based on distance from coast (1: 0–4.99 km, 2: 5–9.99 km, 3: 10–14.99 km, 4: 15–19.99 km, 5: ≥ 20 km). 

Taking into account nymph genus as the external factor and bacterial OTU, genus and family dissimilarity matrix, the ANOSIM histograms revealed significant difference between the unordered tick genera sub-groups (*Ixodes*, *Haemaphysalis*) (OTU: R = 0.340, *p* = 0.003; family: R = 0.353, *p* = 0.003; genus: R = 0.328, *p* = 0.002;) (Figure 6). Similarly, sub-groups based on nymph species as the external factor were significant using ANOSIM (OTU: R = 0.667, *p* = 0.001; family: R = 0.471, *p* = 0.001; genus: R = 0.488, *p* = 0.001) (Figure 6).

When the location of collection was selected as the external factor and bacterial OTU, genus and family dissimilarity matrix, the ANOSIM histograms revealed significant difference between the unordered location subgroups (Mona Vale, Frenchs Forest, Manly, etc.) (OTU: R = 0.340, *p* = 0.001; family: R = 0.234, *p* = 0.001; genus: R = 0.258, *p* = 0.001) in nymph ticks (Figure 6). Similarly, nymph sub-groups with geographical region (Northern Beaches, North Shore, South Coast) as the external factors were significant using ANOSIM (OTU: R = 0.171, *p* = 0.006; family: R = 0.086, *p* = 0.010; genus: R = 0.081, *p* = 0.013), (Figure 6). However, nymph sub-groups with geographical orientation (north or south of Sydney) as the external factors were not significant by ANOSIM (OTU: R = 0.136, *p* = 0.113; family: R = 0.041, *p* = 0.169; genus: R = 0.034, *p* = 0.210) (Figure 6). While the *p*-value recorded for the geographical orientation was not traditionally regarded as significant (*p* > 0.05), the ANOSIM histogram curve still indicates a normal distribution. Sub-groups for nymph ticks with the coastal proximity (1: 0–4.99 km, 2: 5–9.99 km, 3: 10–14.99 km, 4: 15–19.99 km, 5: ≥ 20 km) as the external factors were not significant by ANOSIM at the OTU-level (R = 0.197, *p* = 0.302), but were significant at the bacterial family and genus levels (family: R = 0.126, *p* = 0.007; genus: R = 0.127, *p* = 0.011) (Figure 6).

Taking into account sex as the external factor for adult ticks and bacterial OTU, genus and family dissimilarity matrix, the ANOSIM histograms revealed significant difference between the unordered sub-groups (male/female) (OTU: R = 0.008, *p* = 0.446; family: R = −0.007, *p* = 0.474; genus: R = 0.013, *p* = 0.416) (Figure 6). Sub-groups based on the location of collection for adult ticks as the external factor were significant using ANOSIM (OTU: R = 0.237, *p* = 0.001; family: R = 0.192, *p* = 0.002; genus: R = 0.208, *p* = 0.002) (Figure 6). Similarly, adult tick sub-groups with geographical region (Northern Beaches, North Shore, South Coast, etc.) as the external factors were significant using ANOSIM (OTU: R = 0.173, *p* = 0.001; family: R = 0.123, *p* = 0.005; genus: R = 0.124, *p* = 0.003), (Figure 6). However, among adult *I. holocyclus*, sub-groups with geographical orientation (north or south of Sydney) as the external factors were not significant by ANOSIM (OTU: R = 0.000, *p* = 0.455; family: R = −0.007, *p* = 0.544; genus: R = −0.005, *p* = 0.499) and had no relationship with the microbiota (Figure 6). Sub-groups for adult ticks with the coastal proximity (1: 0–4.99 km, 2: 5–9.99 km, 3: 10–14.99 km, 4: 15–19.99 km, 5: ≥ 20 km) as the external factors were not significant by ANOSIM (OTU: R = 0.058, *p* = 0.112; family: R = 0.048, *p* = 0.141; genus: R = 0.052, *p* = 0.134) (Figure 6).

### 2.7. Absence of Borrelia spp. in Ixodes holocyclus, Ixodes trichosuri, Ixodes tasmani and Haemaphysalis bancrofti from Sydney

The *Borrelia* spp. nested PCR assay at the *16S* rRNA gene was negative for all nymph and adult ticks examined (*n* = 200; nymph: *n* = 100, adult: *n* = 92). The negative controls did not amplify any *Borrelia* spp. DNA. Positive controls were omitted to prevent the risk of any cross-contamination.

Implementing NGS technologies, the pilot study using two *16S* rRNA diversity profiling assays (V1-V3 and V3-V4 hypervariable regions) did not record the presence of *Borrelia* spp. in the adult *I. holocyclus* ticks. Similarly, nymph and adult ticks were free of *Borrelia* spp. at the *16S* rRNA V3-V4 hypervariable region.

## 3. Discussion

This study has shown that in Sydney, NSW, people and their companion animals are exposed to *Ixodes holocyclus*, *Ixodes trichosuri*, *Ixodes tasmani* and *Haemaphysalis bancrofti* nymph ticks, which has been confirmed at the morphological and molecular (*cox*1) levels. It is curious that the species diversity of questing nymphs was notably higher than the species diversity of adult ticks from dogs in veterinary clinics in the surrounding areas, where only *I. holocyclus* was recovered. The presence of only *I. holocyclus* on dogs in veterinary clinics is indicative that it is only *I. holocyclus* that has a paralytic effect on hosts and does not support previously purported claims that *I. trichosuri* may contribute to tick paralysis [29]. Interestingly, a study done in Sydney between January 1990 and December 1992 found that *I. holocyclus* was the most common nymph and adult tick, followed by *Haemaphysalis longicornis* and *H. bancrofti*, which were only confirmed morphologically [16]. Nearly three decades later, *H. longicornis* was not recovered from the environment or from dogs in veterinary clinics, while *I. holocyclus* remains to be the most common tick in the area. This has been the first study to the authors’ knowledge, of this scale, to have flagged for questing nymphs, identified them morphologically and molecularly, and used modern NGS techniques to determine the tick’s bacterial diversity from the Sydney region.

In recent years, NGS technologies has been used to detect and study the presence of pathogenic bacteria and those significant in human and animal health around the world, but few have compared the microbial diversity between tick’s life cycles, especially in Australia [3,5,6]. Further, considering the interest in the detection of *Borrelia* spp. in Australian ticks, few studies have looked into finding *Borrelia* spirochaetes in unengorged Australian nymphs in the past 25 years [16]. In North America and Europe, typically, the nymph stages of *Ixodes scapularis*, *Ixodes pacificus* and *Ixodes ricinus*, are the main vectors for transmitting and infecting humans with *Borrelia burgdorferi* sensu lato (s.l.) [19,30,31]. The *Borrelia* spirochaete population within the nymph tick is amplified following the larva-to-nymph moult and is higher in unfed nymph ticks compared to unfed adult ticks [25,32]. It has been found that nymph density and *B. burgdorferi* s.l. infections were positively correlated, suggestive of the importance of the role of nymph ticks as a vector [33,34,35]. Hence, to accurately determine the possibility of a local infection of Lyme disease in Sydney, it was crucial that nymph ticks of the *Ixodes* genus were screen for the causative agent, *B. burgdorferi* sensu lato.

While *Borrelia* spp. has been detected in Australian ticks in Sydney, *B. burgdorferi* s.l. had not been identified in Sydney, Australia [2,16]. In 2017, Panetta et al. [2] found a reptile associated *Borrelia* spp. from the goanna tick, *Bothriocroton undatum* Fabricius, 1775, in Sydney by sequencing both *16S* rRNA hypervariable regions, V1-V3 and V3-V4, and confirmed the presence of *Borrelia* spp. in *B. undatum* using a *Borrelia* specific nested PCR. The reptile associated *Borrelia* detected by Panetta et al. [2] was evolutionarily distinct from the monophyletic clade for *B. burgdorferi* s.l., the causative agent of Lyme disease, and it only clustered with other reptile associated *Borrelia*. Our studies utilised the same NGS technologies and nested PCR assay and did not detect any *Borrelia* spp. in our tick samples. In one other Sydney-based study, the midgut from 10,970 ticks (2379 nymphs, 528 males, 1832 females) was dissected out and examined by dark field microscopy for spirochaetes and was cultured using *B. burgdorferi* culture media (BSKII) over three months [16]. Spirochaete-like objects were found, but it was determined that these were not *Borrelia* spirochaetes. Additionally, 1038 ticks (576 nymphs, 62 males, 396 females) were subject to molecular screening for *B. burgdorferi* s.l. using three *Borrelia* specific primer sets, which targeted different regions—outer surface protein A (*ospA*), flagellin (*fla*) and *Borrelia 16S* rDNA that had failed to detect any *Borrelia* DNA in the samples. Utilising modern NGS technologies, Panetta et al. [2] detected reptile associated *Borrelia*, which could have been missed by Russell et al. [16], as the primers at the time may not have been able to pick up reptile or monotreme associated *Borrelia*. Especially considering the presence of the echidna tick, *Bothriocroton concolor* Neumann, 1899, which a monotreme associated *Borrelia*, *Candidatus* Borrelia tachyglossi, has been detected from previously [36]. It was necessary for our study to utilise NGS technologies and the *Borrelia* specific nested PCR to screen for *Borrelia* spp. in our samples. However, this study did not find any *Borrelia* spp. in the flagged nymphs from Sydney, or the adult *I. holocyclus* on dogs in the surrounding areas. For over 25 years, there has been a lot of debate surrounding the capacity for *I. holocyclus* to vector the causative agent of Lyme disease, *Borrelia burgdorferi* s.l. [37]. While we cannot comment on the vector capacity of *I. holocyclus* with regards to *Borrelia* spp., we confirm other similar findings that there has not been any tangible evidence suggesting that classical Lyme disease can be contracted locally, as *B. burgdorferi* s.l. has not been found in Australia to this date [38,39].

Like most other living organisms, ticks require bacteria for their survival and success [40]. These bacteria can be pathogenic or symbionts, and some pest insects, like aphids, have a nutritional dependence on the symbiont *Buchnera* [41]. In the soft tick, *Ornithodoros moubata*, Murray, 1877, one *Francisella* strain, *Francisella* F-Om, has been reported to be an obligate nutritional mutualist symbiont which enables *O. moubata* to feed on vertebrate hosts, and was necessary for the life cycle [42]. Further, *Coxiella* is a known symbiont, particularly in Ixodid ticks, and studies have detected *Coxiella* in *Rhipicephalus sanguineus* and *Rhipicephalus* (*Boophilus*) *microplus*, Canestrini, 1888, from different countries [43,44,45]. Further, while the purpose of *Candidatus* Midichloria mitochondrii (CMM) is still unknown in ticks, it is known to have an intra-mitochondrial lifestyle and appears to play a role in the biology of female *I. ricinus* [46]. It has been purported that CMM may have some relationship with ticks as a nutritional mutualist, and it is thought that it is linked to the tick’s capacity as being a vector for disease [40].

The main benefit of NGS technologies, is the ability to gain rapid insight into the *16S* rRNA bacterial endosymbionts of ticks [47]. One study using pyrosequencing to determine the bacterial diversity in *Rhipicephalus microplus* Canestrini, 1888 found shared bacterial genera among *R. microplus* collected from various countries, implying there is a core microbiota associated with the tick species [45]. The core microbiota of ticks is the known microbial symbionts within tick species. It is expected to be unique for each tick species but common among ticks of that species, and the core microbiota includes symbionts which have ecological or biological functional roles [40,48,49]. Geography and sex can influence the core microbiota of ticks [50,51,52]. It is known that while CMM is present in the reproductive tissues of female *I. ricinus*, and it is absent from *I. holocyclus* [46,53]. The ovarian tissues of all female *I. ricinus* are known to be dominated by CMM, which forms part of the core microbiota [46,54,55,56,57]. It was found that CMM are lacking in the midgut of *I. ricinus*, and the midgut did not reflect the presence of a core microbiota and was heavily influenced by host-blood meal [54]. In *I. holocyclus* ticks, *Ca*. Midichloria sp. Ixholo1 and *Ca*. Midichloria sp. Ixholo2 are found as endosymbionts [53]. Further, *Ca.* Midichloria sp. Ixholo1 and *Ca.* Midichloria sp. Ixholo2 have been isolated from *I. holocyclus* ovaries [53]. This, and the abundance of *Ca.* Midichloria sp. Ixholo1 and *Ca.* Midichloria sp. Ixholo2 in *I. holocyclus*, supports the finding that they are part of the core microbiota of the ticks from this study. From the species clustering seen in our microbial analyses, it implies that the ticks in Sydney do have a distinct, core microbiota, which appears to be unique to the species observed. Microbial overlap is apparent, with *Ca.* Midichloria sp. Ixholo1 and *Ca.* Midichloria sp. Ixholo2 found indiscriminately across all four tick species in the study, hence, *Ca.* Midichloria spp. are obligate symbionts of the endemic Australian ticks in this study and are not exclusive to *I. holocyclus* [15,53]. Ticks in Sydney during 2017–2018 were dominated by *Ca.* Midichloria sp. Ixholo1 (69.41%) and *Ca.* Midichloria sp. Ixholo2 (21.45%), so it is part of the core microbiota of ticks in the North Shore and Northern Beaches in Sydney, NSW. Additionally, this study reports the presence of a previously undescribed bacterial taxon, OTU_2090. The unique taxon, OTU_2090, which had 97.3% identity to *Ca.* Midichloria sp. Ixholo2 (FM992373) found in *I. holocyclus* nymph and adult ticks only. It was absent from *I. trichosuri*, *I. tasmani* and *H. bancrofti* nymph ticks, despite sharing the same environment. From this, we conclude that OTU_2090 is a novel *Candidatus* Midichloria sp., which is unique to and is part of the core microbiota of *I. holocyclus* ticks. Other common tick endosymbionts including *Coxiella*, *Francisella* and *Rickettsia* were absent from our ticks [40,43,44,47]. Like other ticks within the *Ixodes* genus, *Ca.* Midichloria spp. remains to be the dominant bacterial genus in our *I. holocyclus*, *I. tasmani* and *I. trichosuri* specimens [15,58]. Notably, this study confirms the presence of *Ca.* Neoehrlichia arcana and *Ca.* Neoehrlichia australis in *I. holocyclus* nymphs and reports the presence of *Ca.* Neoehrlichia australis in an *I. trichosuri* for the first time. The presence of the *Ca.* Neoehlichia spp. in nymph ticks only highlights the significance of nymph ticks as vectors for disease. Previous studies [59] determined that *Ca.* Neoehrlichia arcana and *Ca*. Neoehrlichia australis was closely related to the *Candidatus* Neoehrlichia mikurensis, the causative agent of Neoehrlichiosis in humans [60]. As nymph ticks feed, host and tick bacterial exchange occurs during the uptake of the blood meal, which is an explanation for the absence of *Ca*. Neoehrlichia spp. from adult ticks in our study. Previous findings have shown that host species affects the tick microbiota [8,61]. The absence of *Ca.* Neoehrlichia arcana and *Ca.* Neoehrlichia Australia in our adult tick samples highlights the importance of screening nymph ticks as vectors for disease. While engorged adult ticks still may carry zoonotic bacteria, our findings are suggestive that the non-commensal bacteria leave the tick host during the nymph tick’s blood meal.

Previous studies in North America and Europe have demonstrated that the location of the collection site [5,52,62,63], sex [4,51,52,64], life stage [5,62] and tick species [52,63] had a strong influence on the diversity of the bacterial taxa within ticks. This study has shown that biotic factors including nymph genera and species, geographical location of collection and geographical region of collection, have an influence on the tick’s microbiota. Like other studies, our study found that the tick species was a factor which effected the bacterial taxa [52,63]. While other studies have compared location of collection, they were based on distances hundreds of kilometres apart [5,52,62,63], this study compares nymph ticks at a much finer scale within a distance of approximately 28 km on the North Shore and Northern Beaches regions of Sydney, Australia. Many collection sites were < 1 km apart, and most collection sites were not separated by more than 10 km. Despite the proximity, our study determined that two geographical factors—location and region—had an influence on the tick’s microbiota. One other study [51] has compared collection site at a similarly small scale, whereby the ticks were collected from the Ames Plantation in Western Tennessee, USA, which determined that soil type influenced the microbiota, however it was stated that this was “possibly due to contamination”. As the ticks recovered in this study are known to parasitise humans and companion animals, the community in the Northern Beaches and North Shore regions in Sydney, Australia are exposed to different bacteria of unknown pathogenicity, which varies depending on the geography and tick species [1,18]. Other factors which affected the bacterial profile of ticks elsewhere included sex, life stage and host species was not reflected in our study [7,52,65]. To eliminate the potential bias for host species to affect the microbiota of the ticks, all adult ticks were collected from dogs in veterinary clinics and all nymph ticks were sourced from the environment. The microbial contents for adults and nymphs were analysed separately, however, Shannon’s and Simpson’s indices indicated there were no significant difference between the life stages. Unlike other microbial studies comparing between at least two life stages, we did not find this to be a significant biotic factor [5,49,51]. While other studies [4,51,52,64] have determined there was a difference in the microbiota of male and female adult ticks, we could not make this comparison due to the lack of male specimens in our study. Further, our study could not make a direct comparison between unfed adult and nymph ticks due to the absence of flagged adult ticks. Flagging for adult ticks could see an increase in the number of male ticks, which would address potential differences between the adult male and female tick microbiota in Australia. Future studies would benefit from flagging for adult ticks to determine the impact of the host-blood meal on the microbiota of ticks.

The decision to employ a pilot study to determine the best method for DNA isolation, and the best hypervariable region to obtain the *16S* rRNA gene amplicon profile for our ticks enabled us to find the optimal combination of DNA isolation method and target hypervariable region for the downstream applications for nymph and adult ticks in Sydney. Other studies have shown that using different DNA isolation protocols can impact the bacterial communities generated for microbiota analyses [2,66,67]. In the study by Panetta et al. [2], the use of two DNA isolation protocols and *16S* bacterial profiling target regions (V1-V3 and V3-V4) found that the V1-V3 hypervariable region and Method 1 (ISOLATE Faecal DNA Kit, Bioline, Eveleigh, Australia), afforded the greatest capacity to detect *Borrelia* spp. in their samples. However, the V3-V4 hypervariable region is favoured as it is associated with having the highest diversity estimates in the sequenced bacterial communities [63]. Thus, analysing different DNA isolation protocols and hypervariable regions was necessary in our pilot study to determine which was the ideal combination to determine the most complete rigorous bacterial profile for the nymph and adult ticks in Sydney, NSW. As we did not determine a significant difference between our chosen DNA isolation protocols, due to the lower number of unassigned OTUs, the V3-V4 *16S* rRNA hypervariable region with the ISOLATE II Genomic DNA Kit (Bioline, Eveleigh, Australia) was determined to be the ideal combination to determine the tick’s bacterial diversity in this study.

## 4. Materials and Methods

### 4.1. Tick Specimens

A total of 159 engorged and un-engorged adult ticks were collected from veterinary practices from the Northern Beaches area of Sydney to the South Coast region of New South Wales for this study between the Australian 2016–2017 summer season (November 2016 to February 2017) (Supplementary Table S1). All ticks were forcibly removed by veterinarians or veterinary nurses at the veterinary practices using one of the following methods: Tick Twister® (O’TOM, Lavancia-Épercy, France), tapered tip forceps or removed manually. After removal, ticks were immediately placed in 96–100% ethanol and stored at −20 °C. Ticks were grouped according to host animal and the host species, number of ticks per host, location of host and date of collection were obtained for accuracy.

A total of 148 unfed nymphs were collected from the Northern Beaches and North Shore areas of Sydney and 27 unfed nymphs and 20 unfed larvae were collected from the South Coast region of New South Wales for this study between July and September 2017 (Figure 1, Supplementary Table S1). All larvae and nymphs were collected using a tick flagging/dragging method, whereby a 1 m × 0.7 m white towel was dragged through shrubs or bush and was visually inspected every 5–10 metres. Ticks which were present were immediately removed using forceps and placed into a 1.5 mL microcentrifuge tube with 96–100% ethanol and later stored at −20 °C. Ticks were grouped according to flagging site and the number of ticks per site, GPS co-ordinate of site and date of collection were obtained for accuracy.

### 4.2. Morphological and Molecular Identity of the Same Ticks Using Two Different Methods

**Figure 7:**
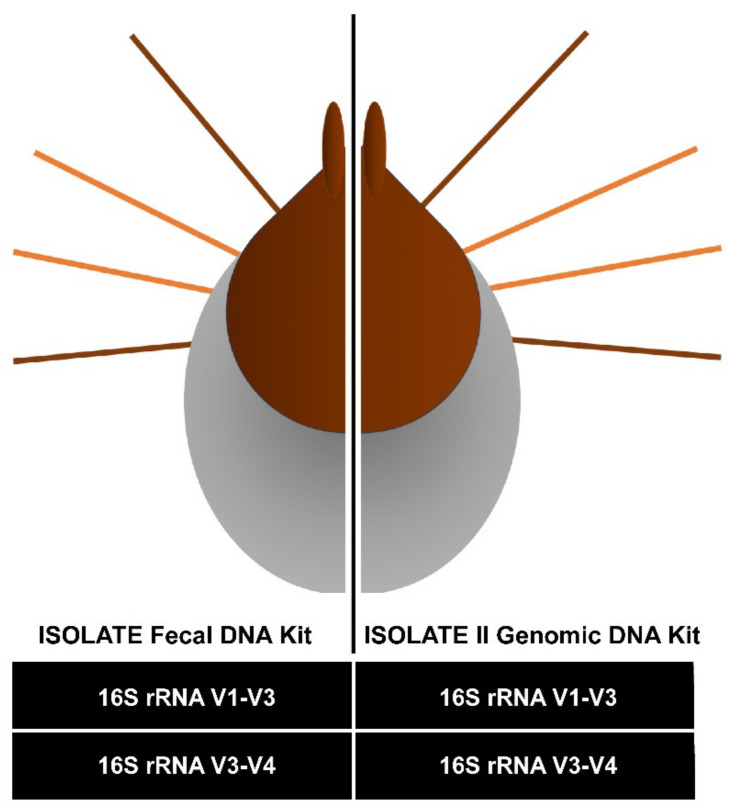
Workflow for the DNA isolation and diversity profiling methods used for the pilot study. Female adult *Ixodes holocyclus* ticks were longitudinally bisected and each half was subject to different DNA isolation protocols—ISOLATE Faecal DNA Kit or ISOLATE II Genomic DNA Kit. The DNA was then subject to sequencing using gene diversity profiling assays, targeting two *16S* rRNA hypervariable regions—V1-V3 and V3-V4.

As a pilot study, six female ticks (SC0063-1 to SC0063-6) morphologically identified as *I. holocyclus,* using keys and guides [17,18], were of varying levels of engorgement and selected from a single host (‘Bear’, German Shepherd Dog, *Canis lupus familiaris*). All ticks were bisected longitudinally using a new, sterilised no. 15 scalpel blade. Each half was subject to different DNA isolation methods, using the ISOLATE Faecal DNA Kit (Bioline, Eveleigh, Australia) or the ISOLATE II Genomic DNA Kit (Bioline, Eveleigh, Australia) (Figure 7). One half was used for faecal DNA isolation, in accordance with the ISOLATE Faecal DNA Kit (Bioline, Eveleigh, Australia) using a mechanical disruption method where the specimen and 5 µL of DNA Extraction Control (Bioline, Eveleigh, Australia) were placed in a supplied 1.5 mL bead-beater with ceramic beads and homogenised with a high-speed benchtop homogeniser (FastPrep-24, MP Biomedicals, Seven Hills, Australia) for 40 s at 6.0 m/s, followed by isolation as per the manufacturer’s instructions. The other half of the tick was used for genomic DNA (gDNA) isolation, in accordance with the ISOLATE II Genomic DNA Kit (Bioline, Eveleigh, Australia) with the following changes: tick samples were digested in 180 µL of lysis buffer, 25 µL of proteinase K and 5 µL of DNA Extraction Control (Bioline, Eveleigh, Australia) for 12–16 h in 56 °C on a heat block.

### 4.3. Morphological Identification and DNA Isolation of Ticks

All ticks examined under a stereo microscope (SMZ-2B, Nikon, Rhodes, Australia) and the species and sex were recorded and identified morphologically with the aid of keys and descriptions [17,18]. Adult ticks were weighed on an analytical balance and the individual weights were recorded.

All tick specimens were surfaced sterilised prior to DNA isolation. Surface sterilisation involved sequential 1 mL washes with a vortex for 1 min in 3% hydrogen peroxide (H_2_O_2_), two 30 s washes in 70% ethanol (*w/v*), 2 min in phosphate buffered saline (PBS, pH = 7.4) and dried on a Kimwipe (Kimberly-Clark, Ingleburn, Australia) [8].

Nymphs were quadrisected using new, sterile no. 15 scalpel blades and whole tick genomic DNA (gDNA) was extracted in accordance with the ISOLATE II Genomic DNA Kit (Bioline, Eveleigh, Australia), and the modifications made as above. The DNA isolation method was completed as per the kit instructions and total DNA was eluted into 80 µL of elution buffer (Tris buffer, pH = 8.5, preheated to 70 °C). Adult ticks were cut into 1-2mm pieces using a new, sterilised no. 15 or 24 scalpel blade and up to 150 mg tick tissue was used for whole tick gDNA isolation, as above.Eluted whole tick gDNA was stored at −20 °C prior to molecular analysis. As DNA isolation kits contribute bacterial contaminants that can impact microbiome analyses [68], two ‘BLANK’ DNA extraction control reactions were included.

### 4.4. Amplification of the Tick Mitochondrially Encoded cox1 Gene

A 604 nucleotide (nt) 5′ fragment of the cytochrome c oxidase subunit I (*cox*1) was amplified using the following primer pairs: LCO1490 (F1) (5′-GGT CAA CAA ATC ATA AAG ATA TTG G-3′) and HCO2198 (R1) (5′-TAA ACT TCA GGG TGA CCA AAA AAT CA-3′) [69] or (F2) (5′-TAC TCT ACT AAT CAT AAA GAC ATT GG-3′) and S0726 (R2) (5′-CCT CCT CCT GAA GGG TCA AAA AAT GA-3′) [70]. 

MyTaq^TM^ Red Mix (Bioline, Eveleigh, Australia) was used for *cox*1 amplifications in 30 µL reactions using 2 µL of template DNA. The PCR cycling conditions were as follows: 95 °C for 1 min, 35 cycles of 95 °C for 15 s, 55 °C for 15 s and 72 °C for 10 s followed by 72 °C for 5 min. All reactions had a positive control, and PCR-grade water was as a no template control. DNA from *Rhipicephalus sanguineus* s.l. was used as the positive control for all reactions. All conventional PCR reactions were conducted in an Applied Biosystems Veriti^TM^ Thermal Cycler (Thermo Fisher Scientific, North Ryde, Australia) or a T100^TM^ Thermal Cycler (BioRad, Gladesville, Australia). PCR products were sequenced at Macrogen Ltd. (Seoul, South Korea). The PCR products of 20 nymphs were bi-directionally sequenced, while the remaining 87 were uni-directionally sequenced.

### 4.5. Tick DNA Sequence Analysis and Phylogeny

Sequences were assembled using CLC Main Workbench 6.8.1 (Qiagen, Vedbæk, Denmark). Phylogenetic analysis of nymph tick DNA and the composition of the nucleotide sequences were determined using MEGA 7.0 [28]. Phylogenetic comparison was made between available *Ixodes* spp. and *Haemaphysalis* spp. tick haplotypes with *Bothriocroton* spp. tick haplotypes as an outgroup from GenBank (National Center for Biotechnology Information, NCBI) using MEGA 7.0 to determine the haplotypic diversity within the Australian tick population [28]. The evolutionary history was inferred using the maximum likelihood (ML) and minimum evolution (ME) method and distances were computed using the Tamura-3 and Kimura-2 methods, respectively, in MEGA 7.0 [28,71,72].

### 4.6. Detection of Borrelia spp. Spirochaete Amplifying a 16S Ribosomal RNA Gene Fragment

A ~1250-nt fragment of the *16S* ribosomal RNA (rRNA) was amplified in a conventional nested-PCR, using previously published primers [36]. MyTaq™ Red Mix (Bioline, Eveleigh, Australia) was used for *16S* rRNA amplification of *Borrelia* spirochaetes in 30 µL reactions using 10 pmol of each primer (Bor-16F (S0778), 5′-TGC GTC TTA AGC ATG CAA GT-3′/Bor-1360R (S0779), 5′GTA CAA GGC CCG AGA ACG TA-3′ for the first round; Bor-27F (S0780), 5′-CAT GCA AGT CAA ACG GAA TG-3′/Bor-1232R (S0781), 5′-ACT GTT TCG CTT CGC TTT GT-3′ for the second round [36]), template DNA (2 µL of DNA for the primary reaction and 1 µL aliquot of the primary reaction PCR product in the secondary reaction), and PCR-grade water. The nested PCR was run with the cycling conditions as described by Panetta et al. [2]. All reactions contained PCR-grade water as a no template control, but a positive control was omitted to minimise contamination.

### 4.7. Amplification and Analysis of the Tick Microbial Profile

As a pilot study, the gDNA and faecal DNA isolated using two different methods from the same six female *I. holocyclus* (SC0063-1 to SC0063-6) underwent diversity profiling of the *16S* rRNA gene at the Australian Genome Research Facility (AGRF, Melbourne, Australia). Tick samples (*n* = 24) underwent DNA quality control (QC) screening via PCR and indexing fluorometry at AGRF (Melbourne, Australia) prior to the diversity profiling at the *16S* rRNA primer targets. All tick samples (*n* = 24) passed QC. Two target *16S* rRNA hypervariable regions, V1-V3 and V3-V4 were sequenced on the Illumina MiSeq (300-nt paired end reads) using the following assays: *16S* (V1-V3): 27F (5′-AGA GTT TGA TCM TGG CTC AG-3′) with 519R (5′-GWA TTA CCG CGG CKG CTG-3′) and *16S* (V3-V4) 341F (5′-CCT AYG GGR BGC ASC AG-3′) with 806R (5′-GGA CTA CNN GGG TAT CTA AT-3′). Paired end reads were assembled using PEAR (version 0.9.5) and the primers were identified, trimmed and processed as previously described by Panetta et al. [2].

### 4.8. Multivariate Statistical Analysis of the Pilot Data for the Single Host Tick Microbiota

Multivariate statistical analyses were used to elucidate patterns of variation within the bacterial composition of the pilot data of *Ixodes holocyclus* (*n* = 6) from a single dog host. The microbiota abundance matrix, OTU taxonomy, and sample factors were imported as metadata for the multivariate analysis in PRIMER v.7 (PRIMER-e, Albany, New Zealand).

The pilot data from the *16S* rRNA V1-V3 (*n* = 12) and V3-V4 (*n* = 12) hypervariable regions were analysed separately. Each tick samples were associated with the following sample factors: tick species ID, tick specimen ID, year of collection, host ID, DNA isolation kit used, and MiSeq sequenced *16S* rRNA gene hypervariable region. To clean the data, initially, unassigned OTUs, mitochondria and chloroplasts were excluded from the analyses. The lowest 5% of the data matrix of OTUs and taxonomy abundance was removed manually.

The V1-V3 *16S* rRNA gene diversity profiling assay yielded 1,246,668 raw reads that were filtered into 789,733 high quality reads (min. 3,743; max. 121,393; *n* = 12). This was clustered into 1642 OTUs and after internal quality control, it was filtered down to 68 OTUs. The V3-V4 *16S* rRNA gene diversity profiling assay yielded 706,769 raw reads that were filtered into 630,336 high quality reads (min. 2149; max. 119,099; *n* = 12). This was clustered into 313 OTUs and after internal quality control, it was filtered down to 64 OTUs. Internal quality control for both hypervariable regions involved manually removing mitochondria, chloroplasts and unassigned OTUs and the bottom 5% of OTUs.

The cleaned data was then imported as metadata into PRIMER v.7 (PRIMER-e, Albany, New Zealand), and was then standardised (samples by total) and fourth root transformed. Bray-Curtis dissimilarity was utilised to measure the variation in bacterial composition within the ticks. Non-metric multi-dimensional scaling ordination, nMDS [73], was used to view the trends of bacterial community similarity between all samples. The goodness-of-fit from the two-dimensional nMDS plot was measured with Kruskal’s stress formula I [73]. Visualisation of the two-dimensional nMDS plots were enriched by annotating the different sample factors (e.g., species of tick, DNA isolation kits) overlaid on to the ordination plots using symbols to assess their possible impacts on the composition of the bacterial communities. Analysis of similarities, ANOSIM [74] (significance level, *p* = 0.05), was applied to test the null hypothesis of no difference between the bacterial communities.

The Mann-Whitney Test for significance was employed on Prism 8 (GraphPad Software, San Diego, USA) to determine whether the bacterial communities generated from the *16S* rRNA hypervariable regions tested (V1-V3 *versus* V3-V4) were significantly different from each other.

### 4.9. Multivariate Statistical Analysis of the Adult and Nymph Tick Microbiota from Sydney

Following the pilot study, multivariate statistical analysis was used to determine the patterns of variations within the bacterial composition of the adult and nymph ticks from the Sydney region, and the south coast of NSW as an outgroup. The microbiota abundance matrix, OTU taxonomy, and sample factors were imported as metadata for the multivariate analysis in PRIMER v.7 (PRIMER-e, Albany, New Zealand).

The data from the *16S* rRNA V3-V4 (*n* = 187) hypervariable regions were analysed separately. Each tick sample was associated with the following sample factors: tick genus, tick species, tick specimen ID, location of collection, region of collection, orientation, coastal proximity (1–5 scale), sex, weight (adult), DNA isolation kit used, and MiSeq hypervariable region. The sample factor orientation was determined to be whether the tick was found towards the north or the south in relation to Sydney. The sample factor coastal proximity was an assigned number from one to five depending on the location of the tick in relation to a coastal body of water (1: 0–4.99 km, 2: 5–9.99 km, 3: 10–14.99 km, 4: 15–19.99 km, 5: ≥ 20 km). A coastal body of water was defined as coastal waters, rivers, lakes, lagoons, undeveloped headlands, marine waters and estuary waters [75].

Of the 203 samples including two ‘BLANK’ DNA extraction control reactions, 188 passed the in-house QC protocols at AGRF (Melbourne, Australia), and were processed for further sequencing at the *16S* rRNA V3-V4 hypervariable region. The V3-V4 *16S* rRNA gene diversity profiling assays yielded 30,714,537 paired end raw reads (18.49 Gb) that were quality filtered into 20,198,718 (min. 259; max. 217,937; *n* = 188) high quality reads, excluding singletons, and clustered into 2287 bacterial OTUs. The removal of chloroplasts, mitochondrion, and unassigned OTUs led to 2284 bacterial OTUs. The removal of OTUs where the total sum was less than 1000 reads led to 137 OTUs being retained. The removal of OTUs found in the ‘BLANK’ reactions, except for OTU_1 *Candidatus* Midichloria sp. Ixholo1 and OTU_1948 *Candidatus* Midichloria sp. Ixholo2, led to 116 bacterial OTUs (12,385,614 paired end reads; *n* = 188) being used for the microbial analysis.

To clean the data, initially, unassigned OTUs, mitochondria and chloroplasts were excluded from the analyses. The total sum of the OTUs from all samples were obtained from the data matrix of OTUs and taxonomy abundance, and if the total sum of the reads within an OTU was < 1000, it was manually removed from analysis. Unassigned OTUs, mitochondria and chloroplasts were excluded from the analysis. OTUs that were present in the ‘BLANK’ reactions were also removed, except OTU_1 and OTU_1948, which was *Ca.* Midichloria sp. Ixholo1 and *Ca.* Midichloria sp. Ixholo2, respectively. OTU_1 and OTU_1948 were present in low numbers in the ‘BLANK’ DNA extraction control reactions (JS3467: OTU_1 96 reads, OTU_1948 0 reads; JS3374: OTU_1 100 reads, OTU_1948 19 reads), and as they were important in the downstream application for microbial analysis, they were selectively retained.

The cleaned data was then imported into PRIMER v.7 (PRIMER-e, Albany, New Zealand), and the data matrix was standardised (samples by total) and then was fourth root transformed. Bray-Curtis dissimilarity was utilised to measure the variation in bacterial composition within the ticks. Non-metric multi-dimensional scaling ordination, nMDS [73], was used to view the trends of bacterial community similarity between all samples. The goodness-of-fit from the two-dimensional nMDS plot was measured with Kruskal’s stress formula I [73]. Visualisation of the two-dimensional nMDS plots were enriched by annotating the different sample factors (e.g., species of tick, DNA isolation kits) overlaid on to the ordination plots using symbols to assess their possible impacts on the composition of the bacterial communities. Analysis of similarities, ANOSIM [74] (significance level, *p =* 0.05), was applied to test the null hypothesis of no difference between the bacterial communities.

**Figure 8:**
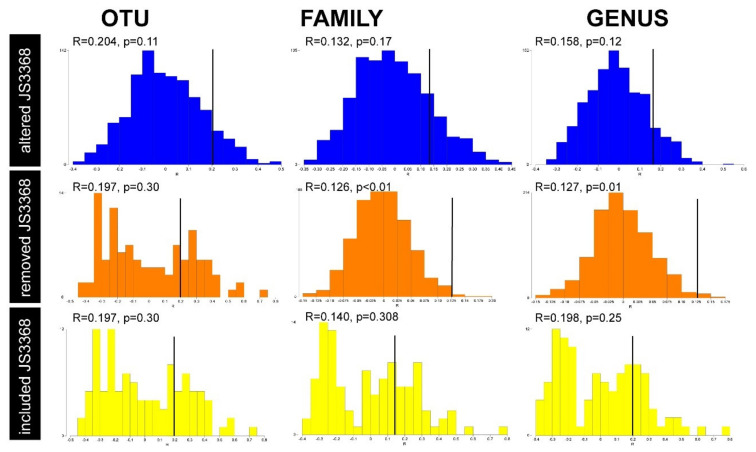
Analysis of similarity (ANOSIM) histograms for the nymph ticks (*Ixodes holocyclus*, *Ixodes trichosuri*, *Ixodes tasmani* and *Haemaphysalis bancrofti*) to rationalise the removal of outlier sample JS3368 from further analyses. Histograms of permutated distribution of the test statistic R (up to 999 permutations), with observed R-values and *p*-values noted at different taxonomic levels (OTU, Family, Genus) evaluated at the coastal proximity biotic factor. Outlier sample JS3299 has been removed from all histograms due to low reads. ANOSIM histograms depicting the artificial altering of coastal proximity factor for outlier sample JS3368 from level 1 (0–4.99 km) to level 2 (5–9.99 km) is shown in blue. ANOSIM histograms depicting the removal of outlier sample JS3368 evaluated at the coastal proximity factor is shown in orange. ANOSIM histograms depicting the inclusion of outlier sample JS3368 evaluated at the coastal proximity factor is shown in yellow.

Multivariable analyses through nMDS revealed that there were clustering of samples based on the nymph tick species identity (Figure 4a). For the nymph ticks alone, two samples (JS3299 and JS3368) were removed from the analyses and were classified as outliers. Sample JS3299 was removed from the analysis as the number of reads following QC was only 259. Sample JS3368 was removed from the analysis as it did not cluster with other *H. bancrofti* nymphs at the OTU level. At the OTU level, the permutation-based hypothesis testing using analysis of similarities (ANOSIM) histograms of permutations were generated for the nymph ticks to determine whether external factors had an influence on the tick’s microbiota. When JS3368 was excluded from the analyses, the ANOSIM histograms revealed that were was not normal distribution at the OTU level (R = 0.197, *p* = 0.302), but the data was normally distributed and significant at the family (R = 0.126, *p* = 0.007) and order (R = 0.127, *p* = 0.011) levels (Figure 8). However, when included in the analyses the data was not normally distributed and was not significant at the OTU (R = 0.197, *p* = 0.299), family (R = 0.140, *p* = 0.308) or genus (R = 0.198, *p* = 0.252) levels (Figure 8). To test it further, the coastal proximity was artificially altered from factor 1: 0–4.99 km to factor 2: 5–9.99 km. This revealed slight non-normal distribution, with the ANOSIM histograms being slightly positively skewed and no significance between coastal proximity and nymph tick’s microbiota at the OTU (R = 0.204, *p* = 0.107), family (R = 0.132, *p* = 0.172) or genus (R = 0.158, *p* = 0.122) levels (Figure 8). From this, we believe the tick is a biological outlier, and could have been translocated artificially during the flagging process, or as a larval tick by the host species movement. It was concluded that both samples, JS3299 and JS3368 would be excluded from further nymph tick analyses as they were characterised as outliers.

### 4.10. Availability of Data

Nucleotide sequence data from this study have been deposited to GenBank (National Centre for Biotechnology Information, NCBI), under the accession numbers MT526908–MT527014 for the nymph *cox*1 sequences, and the SRA database under the BioProject ID PRJNA630349 (https://www.ncbi.nlm.nih.gov/sra/PRJNA630349) for the pilot study, and PRJNA631062 (https://www.ncbi.nlm.nih.gov/sra/PRJNA631062) for the nymph and adult ticks from the North Shore. PRIMER data files and other relevant files are available on LabArchives (https://doi.org/10.25833/x96q-pg77).

## 5. Conclusions

This study has determined that the predominant nymph tick in the Northern Beaches and North Shore communities is the Australian paralysis tick, *Ixodes holocyclus*. Three other endemic tick species were also recorded: *Ixodes trichosuri*, *Ixodes tasmani* and *Haemaphysalis bancrofti*. Notably, in veterinary clinics, only *I. holocyclus* was recorded on dogs. We found that external biotic factors (tick species, geographic location of collection, geographic region, north–south orientation) have a significant impact on the bacterial profile within ticks. The microbial analyses revealed that the tick species in the Northern Beaches and North Shore of Sydney, NSW display a core microbiota, which appears to be unique to each species, with overlap with the bacterial genera. The most common endosymbiont in our ticks are *Candidatus* Midichloria sp. Ixholo1 and *Candidatus* Midichloria sp. Ixholo2. Additionally, a novel endosymbiont, *Candidatus* Midichloria sp. OTU_2090, was present in 96.3% and 75.6% *I. holocyclus* nymphs and adults, respectively. *Candidatus* Neoehrlichia arcana and *Candidatus* Neoehrlichia australis was recovered from *I. holocyclus* and one *I. trichosuri* nymph but was absent from *I. holocyclus* adult ticks.

## Figures and Tables

**Table 1:** Bacterial diversity measures for nymph *Ixodes holocyclus*, *Ixodes trichosuri*, *Ixodes tasmani* and *Haemaphysalis bancrofti*, and adult *I. holocyclus* ticks from Sydney. The following abbreviations are used in the table: *S* total number of OTUs/species; *H*’ Shannon diversity index using the natural logarithm, logarithm to the base e; *1-λ*’ Simpson’s diversity index; SD standard deviation.

	*S*	*H*’ ± SD	*1-λ*’ ± SD
Nymphs	2936	2.938 ± 0.59	0.919 ± 0.07
Adults	2512	2.876 ± 0.82	0.882 ± 0.11
Nymphs + Adults	5448	2.912 ± 0.69	0.903 ± 0.09

## Supplementary Materials

The following are available online at https://www.mdpi.com/2076-0817/9/7/566/s1, Figure S1. Multivariate analyses of pilot study data (V1-V3 vs. V3-V4), Figure S2. Bar graphs for nymph ticks, Table S1. Nymph and adult tick collection information, Table S2. Weight of adult ticks prior to DNA isolation.
